# Supplementary material for: Modulation of miR-29a and ADAM12 Reduces Post-Ischemic Skeletal Muscle Injury and Improves Perfusion Recovery and Skeletal Muscle Function in a Mouse Model of Type 2 Diabetes and Peripheral Artery Disease
Source: Int J Mol Sci. 2021 Dec 31;23(1):429. doi: 10.3390/ijms23010429 (PMC8745107; doi:10.3390/ijms23010429)
Supplement: Supplementary file 1 [file ijms-23-00429-s001.zip › ijms-1452099-supplementary.pdf]

SUPPLEMENTARY FIGURES AND TABLE

Figure S1: Area under curve (AUC) of the intraperitoneal GTT (IPGTT) in unanesthetized mice prior to experimental treatment and HLI surgery

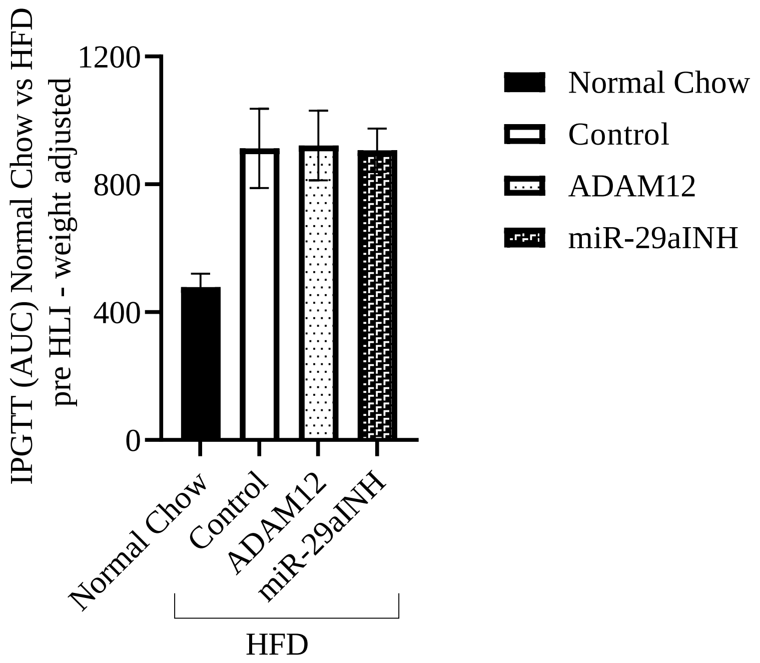

Figure S1: AUC of IPGTT: Glucose tolerance was assessed by intraperitoneal GTT (IPGTT) in unanesthetized mice using 1mg/g mouse of glucose, administered intraperitoneally and glucose measured at: 0, 15min, 30min, 1 and 2 hours. (n=10/group)

Figure S2: miR-29a inhibitor delivered by ultrasound-induced microbubbles did not affect miR-29a expression in the spleen.

A (Day-3)

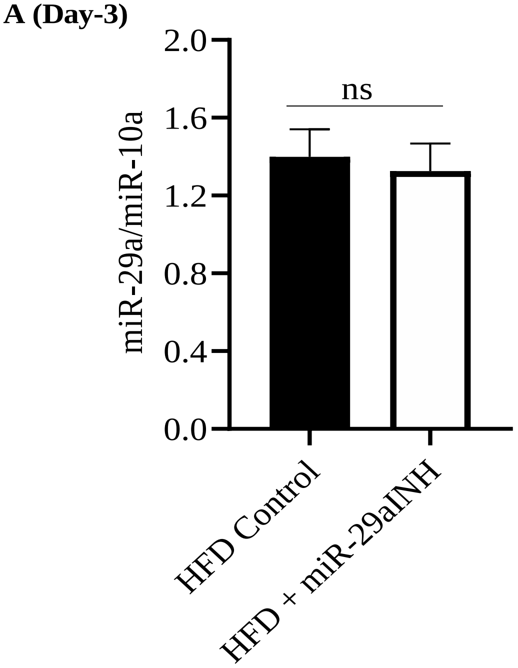

B (Week-3)

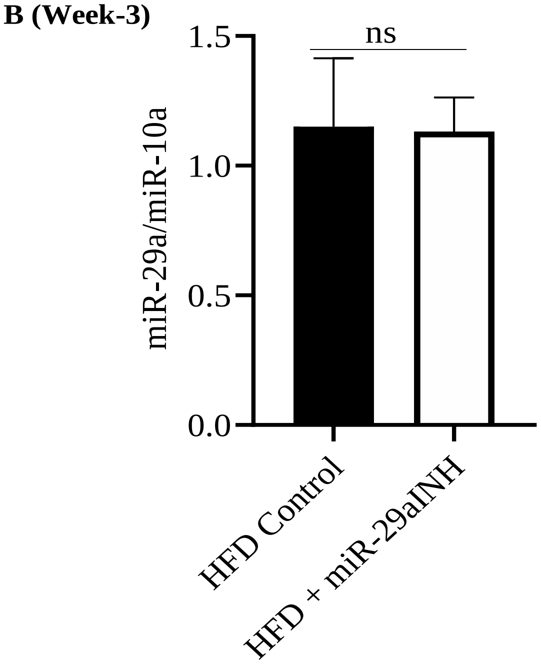

Figure S2: miR-29a levels in spleen lysate show equal expression of control and miR-29aINH treated mice at (A) Day3 and (B) Week 3 post HLI surgery. (n=5/group)

Figure S3: miR-29a inhibitor delivered by ultrasound-induced microbubbles did not affect miR-29a expression in the liver.

A (Day-3)

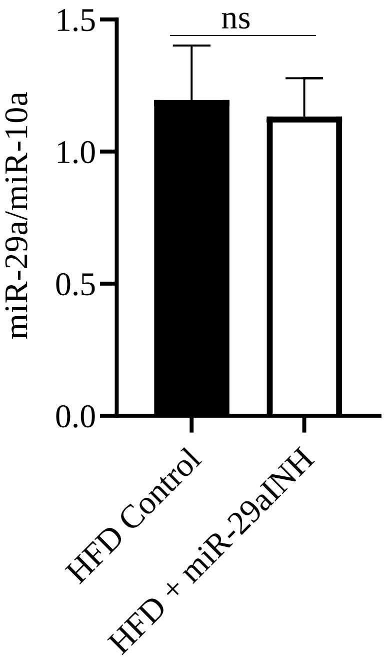

B (Week-3)

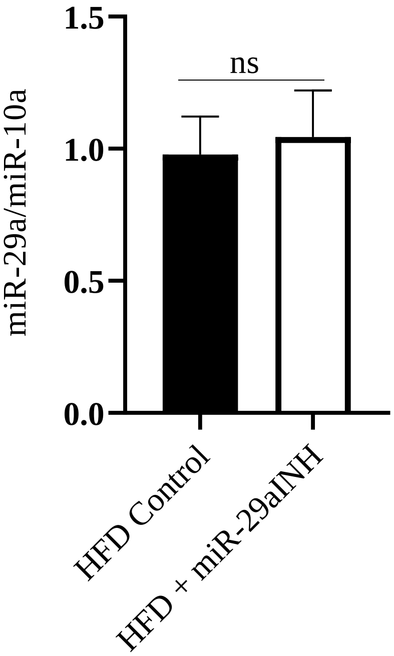

Figure S3: miR-29a levels in liver lysate show equal expression of control and miR-29aINH treated mice at (A) Day3 and (B) Week 3 post HLI surgery. (n=5/group)

Figure S4: miR-29a inhibitor delivered by ultrasound-induced microbubbles did not affect miR-29a expression in the heart.

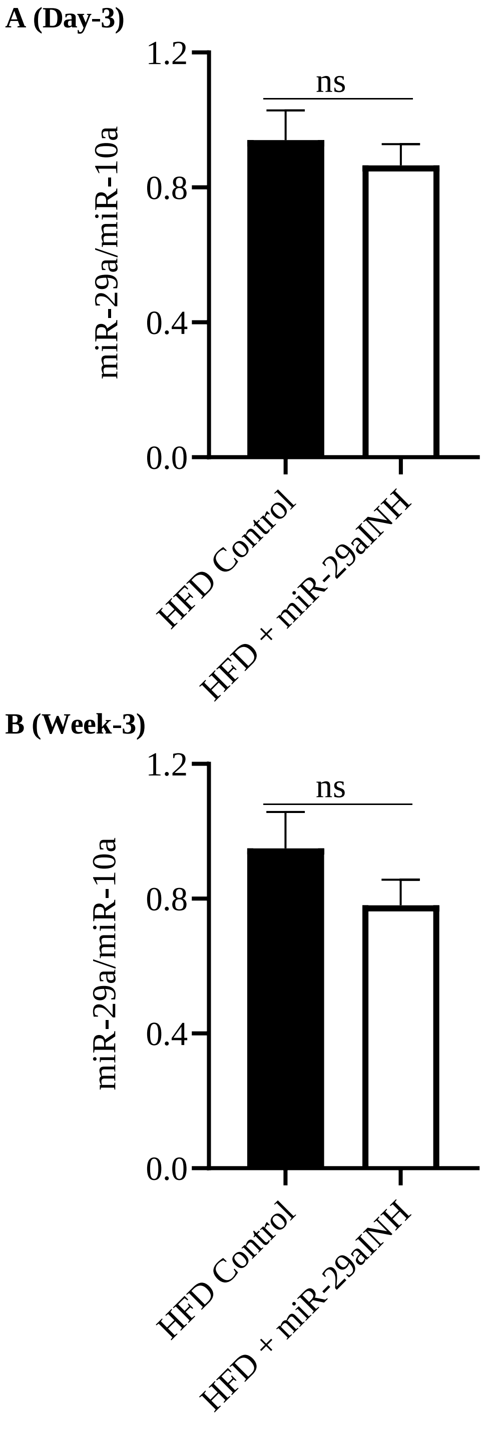

Figure S4: miR-29a levels in heart lysate show equal expression of control and miR-29aINH treated mice at (A) Day3 and (B) Week 3 post HLI surgery. (n=5/group)

Figure S5: miR-29a inhibitor delivered by ultrasound-induced microbubbles decrease miR-29a expression at day3 in the lung but the effect was normalized after 3 weeks.

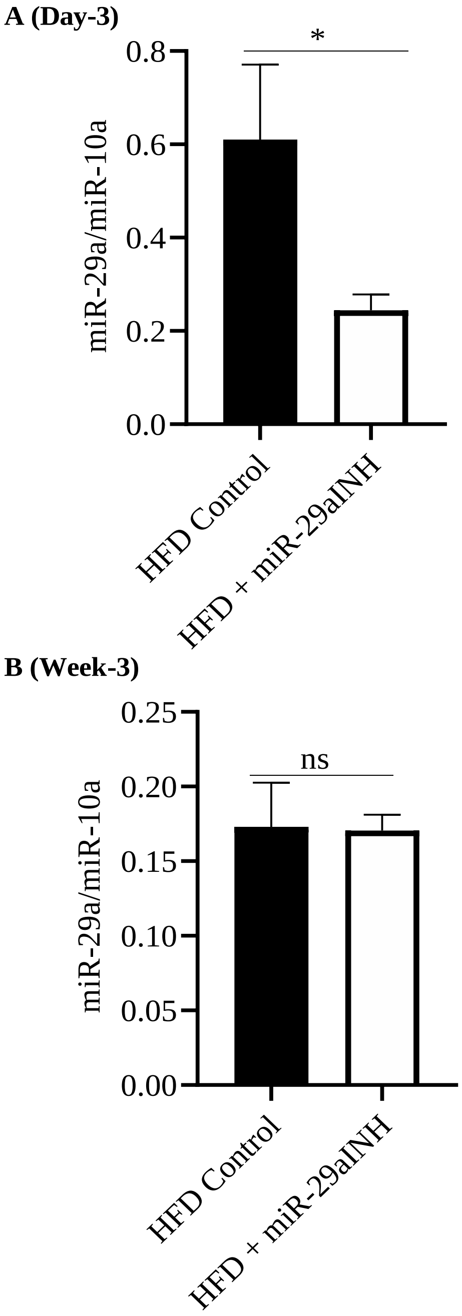

Figure S5: miR-29a levels in lung lysate show decrease expression of miR-29aINH treated mice at day 3, but the effect was not seen after 3 weeks. (A) Day3 and (B) Week 3 post HLI surgery. (n=5/group, *p<0.05)

<u>Figure S6: miR-10a used as a loading control is shown to be stable in ischemic and non-ischemic conditions of normal chow and HFD</u>

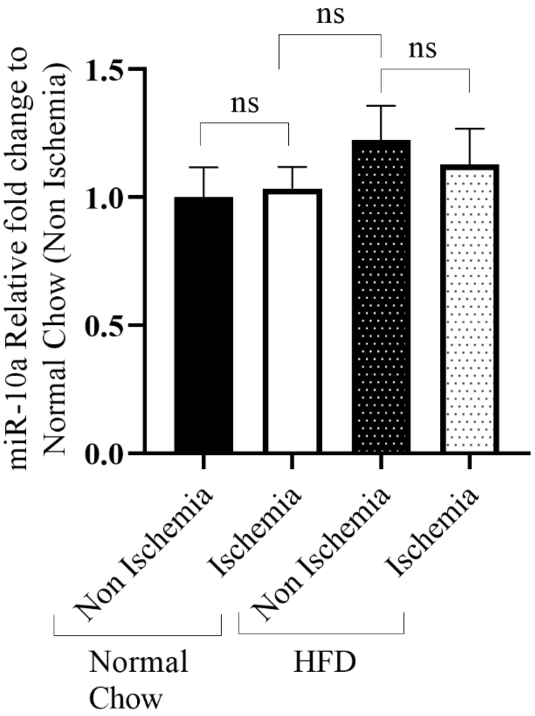

Figure S6: miR-10a levels in muscle lysate is shown to be stable in ischemic and non-ischemic conditions of normal chow and HFD. (n=17/group)

Table S1: Total number of myofiber, $CD31^+$ cells and centralized nuclei counted in ischemic GA

A

**Total number of Myofiber per GA muscle counted**					
		**HFD**			
**Mouse #**	**Normal Chow**	**Control**	**ADAM12**	**miR29aINH**
**1**	8060	4897	5061	6797
**2**	10691	4671	4006	5198
**3**	8758	4390	6590	4371
**4**	4199	5569	6100	10265

B

**Total number of CD31+ cell per GA muscle counted**				
		**HFD**		
**Mouse #**	**Normal Chow**	**Control**	**ADAM12**	**miR29aINH**
**1**	1800	870	1680	2899
**2**	2987	846	1526	3997
**3**	900	772	1699	2098
**4**	1990	917	1983	1996

C

**Total number of Centralized Nuclei per /GA muscle counted**				
		**HFD**		
**Mouse #**	**Normal Chow**	**Control**	**ADAM12**	**miR29aINH**
**1**	120	1266	650	256
**2**	168	1128	726	285
**3**	109	1352	909	215
**4**	60	1106	653	176

Table S1: Total number of myofiber, $CD31^+$ cells and centralized nuclei was manually counted in ischemic GA on day 21 post-HLI. A: Total number of myofiber counted in ischemic GA muscle (n=4/group). B: Total $CD31^+$ cells counted in ischemic GA muscle (n=4/group). C: Total number of centralized nuclei counted in ischemic GA muscle (n=4/group).
